# Barriers to and facilitators of prostate cancer screening among men in Uganda prisons

**DOI:** 10.3332/ecancer.2023.1563

**Published:** 2023-06-22

**Authors:** Innocent Atuhe, Alfred Jatho, Babra Nalwadda, Annabella Habinka Basaza-Ejiri, Lynn Atuyambe, Jackson Orem

**Affiliations:** 1 King Caesar University, PO Box 88, Kampala, Uganda; 2 Uganda Cancer Fund, PO Box 25912, Kampala, Uganda; 3 Uganda Cancer Institute, PO Box 3935, Kampala, Uganda; 4 Makerere University, PO Box 7062, Kampala, Uganda

**Keywords:** barriers, facilitators, prostate cancer, screening, prisons, Uganda

## Abstract

**Background:**

Studies have shown that prostate cancer (PCa) is increasing at a rate of 5.2% per annum in Uganda and as few as 5% of men have ever been screened for PCa in Uganda. The situation may be worse among male prisoners given their ‘vulnerable status’. The goal of this study was to examine the perceptions, attitudes and beliefs of men in Ugandan prisons regarding barriers to and facilitators of PCa screening. This would enable the identification of potential interventional strategies to promote PCa screening among men in Ugandan prisons.

**Methods:**

This study applied the explanatory sequential mixed methods study design. We first conducted 20 focus group discussions and 17 key informant interviews. The qualitative data were analysed to enrich a survey among 2,565 prisoners selected using a simple random sampling technique.

**Results:**

Qualitatively, the belief that all cancers have no cure was a barrier against most participants considering screening to be of any value, coupled with the fear of screening positive for PCa and the associated stress. In addition, poor PCa knowledge and lack of PCa screening services in prisons were perceived as barriers to PCa screening in prison settings.

The quantitative data from the survey of 2,565 participants with a mean age of 50.2 (9.8), indicated that the main barriers to PCa screening were mainly myths, beliefs, lack of screening facilities and technical capacity. The majority believed that creating PCa awareness, conducting screening outreach in prisons, and providing equipment for PCa screening in prisons health facilities will facilitate PCa screening, as well as working with the Uganda prison service to train the prison health staff to perform PCa screen to facilitate Prison Health Centres capacity to screen for PCa.

**Conclusion:**

There is a need to develop interventions to increase awareness among the inmates in the prison health system, while ensuring that the prison health facilities are equipped with the required screening logistics, backed with outreaches from cancer-specialised hospitals/facilities.

## Introduction

Prostate cancer (PCa) is the most prevalent cancer among men in Africa and ranks as the 3rd most prevalent cancer overall with 59,500 incident cases per year (16.4%) of all cancers in men [1–3]. In Sub-Saharan Africa, the PCa burden is relatively higher, representing 20.3% of all cases of cancer in men [2]. Incidence rates are highest in Zimbabwe and Uganda at 38.1 and 37.1 per 100,000 respectively [4], with very poor prognosis [5].

However, this estimate may be low because another study reported the incidence of PCa in Uganda at 65 per 100,000 and a very high mortality-to-incidence ratio of 71% [2]. It is also estimated that PCa incidence in Uganda is increasing by 5.2% annually making it the fastest-rising cancer in the country [6, 7]. In the general Ugandan population, 80% of PCa patients report to health facilities in the advanced stage indicative of low PCa screening rates. Research has also shown that despite the fact that the PCa trend is on the rise, as few as 5% of men have ever been screened for cancer of the prostate [8]; moreover, simple and affordable screening tests are available, despite the conflicting decision on whether and when to use the currently known PCa screening tests [9]. Currently, the known and available PCa screening tests in Uganda are prostate-specific antigen (PSA) and digital rectal exam (DRE) targeting men aged 40 (the most at-risk individuals, especially men with a family history of PCa) or 50 years and above. PSA is a protein produced by cells of the prostate gland and the test measures the level of PSA in the blood. During the DRE procedure, the doctor feels the surface of the prostate gland for bumps, hard spots, and any other abnormalities.

This uptake of PCa screening is a big burden to the health system globally, regionally and Uganda in particular. The situation may be worse among the prisoners given their 'vulnerable status' and the challenge of accessing and providing cancer care behind bars by the inmates and the healthcare workers respectively [10, 11], including restriction to movement of the inmates. This could grossly worsen the extent of the cancer control inequity and the capacity to seek cancer information and screening services. Despite these worrying statistics, research focusing on PCa in Africa in general and Ugandan prisons, in particular, is lacking and many questions about the disease remain unanswered in this setting, including information on barriers to and facilitators of PCa screening in prison settings.

Further, a literature review of both published and unpublished data shows that there is a dearth of information about barriers to and facilitators of PCa screening in prison settings. After conducting a thorough literature search using PubMed, Cochrane and CINAHL databases, we found that there are no published studies that have specifically examined the barriers, attitudes and beliefs of men in Ugandan Prison settings about PCa screening. A few studies on cancer in prison settings have been conducted in Australia, Canada and the United States of America, the most recent being by Olds *et al* [12].

These studies reported limited research on barriers to cancer services access in prison settings and further recommended the need to carry out more studies to effectively elucidate how barriers to good quality cancer care for prisoners may be identified and mitigated. Whether the barriers and facilitators identified in studies in the United States of America, Australia and Canadian prisons are similar or different from those among men in Ugandan prisons is unknown. The motivation for this study is to close the knowledge gap on barriers to and facilitators of PCa screening in Ugandan prison settings. This will enable the Uganda Cancer Institute (UCI) to plan interventions among this vulnerable population.

Therefore, this study aimed to examine the perceptions, attitudes and beliefs of men in Ugandan prisons regarding barriers to and facilitators of PCa screening. It was envisaged that this will enable the identification of potential interventional strategies to promote PCa screening among men in Uganda prisons. This study examined the following specific Aims;

*Specific aim 1*

To identify the perceptions of men in the Ugandan prison setting regarding PCa screening so as to design specific interventions that could improve PCa screening in the prison population.

Under this aim, the study answered the following research questions;

- What do men in Ugandan prisons think about PCa?
- How can PCa be screened and detected early in a prison setting? And,
- What can be done to increase PCa screening among men in Ugandan Prison settings?

*Specific aim 2*

To identify the cultural factors (barriers and motivators) among men in Ugandan prison settings regarding PCa screening so as to design culture-specific interventions that will increase PCa screening uptake for the prison population.

Under this aim, the study answered the following research questions;

- Have men in Ugandan prisons been screened for PCa? If not, what are the reasons? If yes, what are the reasons?
- What myths and beliefs prevent or stop men in Ugandan prisons from getting a prostate checkup? How come men do not get a checkup for their prostate, and how can they be supported to have themselves screened?
- For what reasons would men in Ugandan prisons seek prostate screening services

*Specific aim 3*

To identify the interventional strategies that men in Ugandan prisons believe could help health care providers with promoting and increasing PCa screening.

Under this aim, the study answered the following research questions;

- What is the best way to educate men in Ugandan prisons about PCa?
- What is the best way of helping men in Ugandan prisons to get screened for PCa?

## Methods

### Study design

We utilised the explanatory sequential mixed methods study design [13]. This approach is the most appropriate for studying a phenomenon of which little is known. We began with the qualitative phase where we collected qualitative data, analysed it and then the results were used to further inform the quantitative phase. This way the quantitative variables were logically extracted [14, 15].

### Study population and inclusion criteria

The study population comprised of men in Ugandan prisons. The primary target population of this study was men aged 40 years and above who have stayed in the prison for more than 3 years. The authorities in the Ugandan prisons, policymakers in the Ministry of internal affairs, the Ministry of Health, the Academia as well as the UCI were the secondary audience for this study.

### The qualitative and quantitative methods used

#### Qualitative component

We used qualitative methods to understand prisoners’ beliefs, experiences, attitudes, behaviour and interactions regarding PCa screening to generate non-numerical data. The two main qualitative methods of data collection; focus group discussions (FGDs) and key informant interviews (KII) were employed.

*Focus group discussions*: We conducted FGDs to draw upon participants’ perceptions, attitudes, feelings, beliefs and experiences about screening for PCa in Ugandan prisons. This method allows small group discussions of 6 to 12 participants to convene for over an hour to discuss issues of their interest as well as the researcher’s interest. Participants from rural and urban prisons were purposively sampled based on the capacity to discuss the subject matter. We conducted 20 FGDs from both urban and rural prisons, grouped by prisoners who have stayed for 1–3 years and those that have stayed longer in different age brackets of 40–54, and 55 years and above. This gave us an opportunity to have varying opinions across ages and residencies.

*Key informant interviews*: To further explore the barriers to and interventions for PCa screening, we conducted high-level interviews with key informants (KIs). We planned to interview 30 KIs, however, 17 KIs were interviewed, in the following categories; prison warders, health staff of clinics in the prisons, cancer care facilities as well as policymakers, and technocrats in the ministries of internal affairs, health and Uganda prisons services (UPS). We used a snowball-sampling technique until saturation was reached. Thematic content analysis was done rapidly to enable us to obtain concrete views and variables to measure in the quantitative survey [14, 15].

#### Quantitative component

We used a cross-sectional study design for this survey. Using lists from UPS, we stratified the country according to the major regions (North, East, South, West and Central), and purposively picked the main regional prisons. Then we again purposively selected only men aged 40 years and above in the prisons. At this point, we performed proportionate sampling based on the size of the men above 40 years in the prisons. With a population size of 65,000 prisoners [16], 95.5% are men making 62,075 [16, 17]. Each of the five regional prisons has a population size of about 12,000 prisoners. We determined the sample size using the formula by Kish [18] below, and as guided by Wang [19]:


$$\frac{\frac{Z^{2}\cdot p(1-p)}{e^{2}}}{1+\left(\frac{Z^{2}\cdot p(1-p)}{e^{2}N}\right)}$$

where *n* = sample size, *N* is the population size and *e* is the level of precision at a 95% confidence level and *p* = 0.05. This generated a sample size of 572 per region. Thus, the overall sample size was 2,863; however, this study managed to enrolled a total of 2,565 participants for the quantitative strand. The data were collected using a questionnaire that was informed by the qualitative findings.

### Ethical consideration

Prisoners are a vulnerable population and thus all efforts were taken to ensure that none of their rights was breached. We explained to the study participants the objectives of the study as well as the benefits and risks associated with participating in the study. In addition, we informed them of the right to withdraw at any time of the process in case they feel unconformable. As such a written informed consent (ICs) form was provided. English ICs were translated into other four regional local languages (Luganda, Luo, Runyankole/Rukiga and Lugisu/Lumasaba) spoken in the catchment areas of the sampled prisons to fit the diverse nature of four major tribes in Uganda. For confidentiality, participants were identified only by initials and code numbers. This study was approved by an accredited Research and Ethics Committee at the UCI and the permission to conduct the study was granted by the Uganda National Council for Science and Technology.

### Innovation

The innovation of this study lies in the explanatory sequential mixed methods approach that was used to reach a very vulnerable population and seek to develop an appropriate intervention method to screen for cancer of the prostate. Secondly, understanding the underlying issues that hinder access to screening may also help other vulnerable populations (such as women with cervical cancer) and enhance public health services.

#### Quality control

We trained our research assistants and conducted a pre-test to check for the accuracy of our study instruments. During data collection, we ensured that our field supervisors act as a link to the prisons and investigators. Besides, this being a vulnerable population, we ensured that all ethical standards were observed.

#### Data management and analysis

Qualitative data were transcribed from digital recording equipment and summarised. The FGD and KI transcripts were read several times while making notes in the transcript. A qualitative content analysis technique was applied. Data was condensed without losing quality and meaning. Open coding was done and the codes were grouped into categories and then themes were identified as stipulated by Graneheim and Lundman [20] and Wang [19]. Quantitative data was entered into Research electronic data capture software, a secure web application for building and managing surveys and databases, fitted with a range of consistency checks. The data were then exported to STATA Version 16 for analysis.

## Results

### Qualitative results

#### Men’s perceptions regarding PCa screening

*Perception about PCa:* Generally, there were a number of perceptions that men in prison had about PCa, the majority of the participants perceived PCa as a disease associated with social, economic, and individual characteristics, and not curable, thus a killer disease. In addition, PCa was perceived as a disease that should be treated as a private matter because some of its screening methods intrude on the screenee’s private body parts. Others believed it was a disease for rich men and runs in the family.

**‘** *Prostate cancer has no treatment…… when one has cancer, it is a sure death’* **(FGD_Luzira prison, above 54 years)**

‘*Men in Uganda think that prostate cancer is a killer disease…..it is a disease that once you are diagnosed with it, it may not cure’*** (KII_)**

‘*We understand it as death as there is no treatment and spends a lot of time in pain’.* (**FGD_Mbale Main Prison, 40–54 years)**

‘*I have been here for the last six years, I heard from the medical staff that it (Prostate cancer) affects men from 40yrs and above*… men who *not sexually active are bound to get it’*. (**KII_**)

‘*………Prostate cancer is for the old people who have grown up mostly those who have multiple partners, they move here and there, they can perhaps get the disease’*. (**KII_**)

‘*Prostate cancer is the disease of the rich people who are old’* (**FGD _ Mbale Main Prison, 40–54 years**)

‘*And others think it is genetically acquired, if somebody is not born with it, he will say it is not running in the family so I don’t expect myself to get it’*. (**KII_**)

Besides, some of the inmates had a perception that PCa is associated to an individual lifestyle where they revealed that unhealthy behaviours like cigarette smoking, homosexuality as well as masturbation would expose one to contract PCa. Also, spending a long period of time without having sexual intercourse was perceived to predispose men to PCa.

‘*I think it is because of the situations in the prison. Not having sex, I think it can bring that cancer. Others say that may be the things we use are the ones that cause this cancer’* **(FGD Luzira prison, above 54 years)**

‘*Prostate cancer may be caused by inactive sexual activity’*** (KII_)**

The prison environment in regards to sanitation was also perceived as one of the risk factors of prostate cancer and the food that the prisoners eat was another issue raised by the participants.

‘*I think dirtiness causes cancer, using one public toilet, if we are not clean there is that air that rises from down the toilets and does back fire after someone has been there in the toilets, so if you go there without cleaning it with soap you can also contract cancer’* (**FGD Luzira Prison, 40–54 years)**

‘*Like one kind of food eaten, sometimes we depend on one kind of food like beans and posho and eat them for a long time, for me I think it can bring that situation because they don’t get us greens and fruits to eat’*. (**FGD Luzira Prison, 40–54 years)**

*Perceptions towards early detection of PCa:* Although some participants had a positive perception that early detection of PCa is very important, the majority did not have any value attached to early detection because of a perception that PCa is an incurable disease that would lead to death irrespective of whether it is detected early. Some of the participants urged that early detection would come with worry to lose a loved one or the dependents worrying that soon they would lose the support that they were receiving from one diagnosed with cancer; thus, people end up shunning screening that would perhaps lead to early detection and saves a life.

‘*Most of men in my area where I come from think if checked and found with prostate cancer, they may lose their wives or even those not married will not be accepted by women because women think they are weak for sex’*. (**FGD Kasangati Prison, 40–54 years**)

‘*Prisoners fear to be checked because if found with a prostate cancer they will be isolated in prisons because fellow prisoners will fear them urinating while sleeping together’*. (**FGD Kasangati prison, 40–54 years)**

#### Ways to increase PCa screening

Creating awareness of PCa was mentioned to be an important aspect towards PCa early detection as most of the men in prisons were not knowledgeable about the disease. Even those that knew about it had minimal knowledge of the screening services and how the disease is of public health importance. Suggestions on how awareness would be created included the use of peer educators among inmates who should give basic information on the screening and PCa, use of information, education and communication (IEC) materials ‘*Use of posters and brochures for prisons specifically targeting the people can help in creating awareness’* (**KII)**

In addition, improving the PCa screening services by providing screening equipment in health facilities was also perceived as a way to increase access and uptake to early detection as well as availing trained health providers to offer the screening services. This was reported in almost all prisons visited.

‘*We need equipment and lab reagents for check-up’*. (**KII**)

Conducting outreaches to prisons by experts in cancer screening was also perceived as one way of creating access to early detection of PCa. This was in line with the interviewed health providers who acknowledged that they did not have skills in PCa screening. Therefore, having outreaches conducted by a trained team would increase the chances of men in prison to screen. Similarly, some participants mentioned that there is a need for cancer centres to conduct massive free PCa campaigns that would provide a range of services like creating awareness and screening services.

Also, the formation of a partnership between the UCI and UPS was suggested to ensure that men in prisons are able to access services and this can be enhanced by also going ahead to establish regional screening centres across the country.

‘*Fo*r *me it is okay to screen if at all the prison accepts competent doctors to come and check who has and who does not and it helps us to get early treatment or prevent it early’.* (**FGD Luzira Prison, above 54 years)**

‘*Prisoners who have ever screened and/or checked for prostate cancer should be used to reach their fellow prisoners and advocate for checking prostate cancer and reduce on the myths and beliefs that prevent or stop men from check-up’* (**FGD Kasangati Prison, 40–54 years**)

‘*Prison services should accept Uganda Cancer institute to come here to train trainees inside prisons because even for other diseases like TB, HIV etc., we have counselors who help us here…. If you give them priority and chance then we shall be health-educated and treated’*. (**KII**)

#### Barriers to PCa screening

Overall, there was a minimal number of respondents that reported that they had ever screened for PCa. Screening for PCa was reported to be a new concept in a prison setting.

‘*Prostate cancer is something which is new to most people...*.’ (**KII**)

*Myths and beliefs related barriers to PCa screening:* The general belief that all cancers have no cure was one of the barriers that inhibited most participants not to consider screening as of any value, coupled with fear of being told that one is positive and the stress that comes with it. Participants revealed that they would rather live without getting to know the unknown.

‘*The disease is incurable and kills, so, it’s better not to test so as not to die fast’ (***FGD_ Mbale Main Prison, 40–54 years)**

**‘** *Normally when you send them to go for screening for cancer, they would say. No, we go for Sexually transmitted diseases (STDs). I think people fear cancer and if they know they have cancer they know they are going to die. So, they think the moment you get cancer you are going to die, diagnosis will be poor and then side effects of the drugs is also toxic’* **(KII)**

**‘***The fear of death! If you know that …… if you screen and find that you have it then you know you are going to die. So, somebody will fail to go to screen because he is fearing the unknown’* **(KII)**

**‘***Even when we find a patient who is diagnosed with cancer, they don’t want even their relatives to know, because they know it's going to cause fear among the family members especially if he is the one who was taking care of them. So, it’s better to live with the disease than knowing …. having stress, you know..., that psychological torture if you know you have cancer, it may reduce the time you are living ….is actually a myth’*. (**KII**)

Also, screening for cancer in a hospital setup was considered to be expensive, especially in a private health facility.

‘*I used up to 300,000/ = Uganda shillings which is not possible for everybody’ (***KII***)*

Other than the belief that PCa could be inherited, some participants had a belief that PCa is caused by witchcraft which does not need medical attention, including screening, thus, does not need to screen for.

‘*…some of them belief that, this is something out of bewitching, so there is that school of thought that they are bewitched and end up getting that. However, others think that it’s something that may be hereditary’…* (**KII**)

In addition, engaging in extramarital affairs was another myth that was reported, as well as being a condition that develops as a result of untreated STDs; hence, no need for screening. Besides, the belief that treatment given to cancer patients worsens the health condition of patients and the fear to be stigmatized shun away men from screening. Some participants believed that once one has been diagnosed with PCa they would be rejected by the community and also be divorced by their spouses, thus, better not to screen. Though most prison settings had something when asked to share on the myth and beliefs surrounding PCa screening, men in FGDs from Gulu main prison revealed that they did not have any myths about the screening.

‘*Others think that for example when you get a kid (child) outside or have sex outside your wedlock, you can easily get cancer’*. (**KII_**)

‘*… they know if they screen and found positive, they will be put on treatment, and they end up dying, that is their myth which may not be true technically’* (**KII_**)

‘*No beliefs or myths may stop men from getting prostate cancer checkup. For us in Acholi I have not had of any myth or belief that stop men from screening because for us in Acholi we depend on hospital for treatment’*. (**FGD_ Gulu Main Prison, above 54 years**)

*Other barriers:* Prison health units not being equipped with the necessary resources to facilitate PCa screening services was one of the major barriers to access to PCa screening by men in prisons. The resources that were reported to be lacking included the screening equipment and supplies. However, it was noted that the prison health staff were not skilled to conduct the PCa screening. Still, in line with this, participants revealed that they have not had any outreach or experts in cancer visiting their prisons to offer PCa services.

‘*Knowledge gap among us. We really don’t know what to do. Secondly, we don’t have the equipment …. Personally, I don’t know even the equipment used for PCa screening’*. (**KII)**

‘*There is no any cancer screening service in prison including prostate cancer so, it is better to begin providing such services’*. (**FGD Gulu Main Prison, above 54 years**)

Lack of knowledge of PCa by men in prison was another key barrier to screening for PCa. Although the majority of the men had never heard about PCa, they did not know its risk factors, symptoms, and where screening services can be sought from.

‘*We do not know of this disease…. what could be signs and symptoms of the disease as some people say it is the result of things not known’* **(FGD Mbale Main Prison, 40–54 years)**

‘*It all goes back to information about PCa…it's something which is real if you have not told people that it affects them…inform the population that this is real. So, if I go back to statistics I doubt if 1 out 10 have gone to screen for PCa. It’s all about awareness and this is a disease that can affect anyon*e’. (**KII**)

‘*For us we don’t know the exact reason for this cancer but there are no cancer services in this prison’*. (**FGD Gulu Main Prison, above 54 years**)

Furthermore, the prison schedule and policies were noted as other barriers to PCa screening among men in prison. In the prison setting, the routine activities are either going to court or going out to work, thus, living no opportunity for men in prison to seek for health services as they would want, unless one presents with a medical condition, that is when they are given treatment. This is coupled with security and safety of the inmates; they are not allowed to go for services out of the prison set-up unless a referral and when the condition is critical. Therefore, in regard to PCa screening, this is looked at as not an emergency that necessitates a referral out of the prison setting, especially to men in prison that have committed capital offences.

‘*Security reasons. Some people are having very sensitive cases whereby in case you take him out there then he disappears. The reason why sometimes they are taken to the main prison is because he has a very big case and what you are talking about doesn’t look serious to the Asikari (prison warden/security guards)’'* (**KII**)

‘*As far as Uganda prison is concerned, men have no time to be very sincere. Someone wakes up at 5.45 am ready for work, some of them go to court around that time, buses set off at 6.50 am, they come in the evening very tired and they are unable to access……… so the routine can be a hindering factor’.* **(KII_ Luzira**)

‘*Provide prostate cancer check up in the prison because here we are locked up with no freedom of movement’*. (**FGD_ Gulu Main Prison, above 54 years**)

Similarly, health services in prison setting are more focused on care than prevention or general medical checkup. Men in prisons reported that they access health services only when they are presented with a medical condition that is symptomatic. Thus, services like screening are not conducted, otherwise, screening services for PCa and many others have not been given priority.

’*Health centres accept only prisoners who are sick …. if he doesn’t complain of sickness then doesn’t come to the hospital’.* (**KII)**

Stigma associated with being a cancer patient was another barrier. The male inmates reported that they have not been educated on how to deal with the anxiety and the stigma that comes with one being found with cancer. This makes it harder for one to take the initiative to test even if the services were provided.

‘*Since this is a secret between doctors and prisoners. It’s hard for a cancer patient to come out and say am suffering from cancer, knowing that in the big population he is in, they will segregate him or hate him hence the disease continues to grow’.* **(FGD_ Luzira prison, above 54 years)**

‘*Fear of stigma of having the disease…. there is a lot of anxiety concerned with the disease’* (**FGD_ Mbale Main Prison, 40–54 years)**

On the other side, one KI believed that men in prison are not at risk of developing PCa, since they were not sexually active in the prison. However, this was refuted by one of the inmates who alluded that men in prison were at a high risk of developing PCa due to spending long time without having sexual intercourse. This suggests a low level or even lack of knowledge of PCa in the prison settings.

**‘** *Spending long time without having sex … where would we get the disease’* **(KII)**

Besides, participants further reported that whereas services of other medical conditions have been availed in prisons, especially, HIV, malaria and TB, health providers have not given priority to cancer, including PCa services, thus another barrier to accessing PCa screening services,

‘*I have been in prison over ten years as head of unit Officer-in-Charge of the Prison but have never seen any medical exam of this nature on prostate cancer, it has never been taken seriously in prisons, it is a rare situation’* (**KII**)

*‘Prisoners have not yet been informed that they have to screen as you see how it is on HIV, so many people have not minded about screening for PCa, not that they don’t want, but they are not reached, so even these prisoners have not got informed’* (**FGD_ Luzira prison, above 54 years**)

Fear of the procedure used for screening was another barrier. One participant believed that men have a fear towards the tests that are being used on the private parts since it is one of the body parts considered to be sensitive, and may be associated with causing infertility.

‘*You know being in rural area, there are some beliefs that when you undergo screening and you don’t know what it involves, which part do they use or check? Sometimes people believe that it may make them not to reproduce’* (**KII**)

‘*People don’t believe in testing instruments or anything to be used in areas like private parts or anus’*. (**KII**)

#### Facilitators for PCa screening

On what would be the facilitators to increase PCa screening in the prison setting, the participants suggested that they would have themselves screened if cancer screening, including PCa screening, is established in the prisons while ensuring that there are adequate cancer specialists to handle the patients, providing testing facilities near or within the prisons, and conduct cancer awareness.

Besides, having a screening programme in prison was suggested as a motivator for the inmates to check up for cancer. Some liked the idea of screening to the extent that they suggested having mandatory entry screening of PCa would increase the number of check-ups. Also, doing follow-ups to seek screening to the age group that is more likely to be at risk of developing PCa was suggested as a facilitator to increase on the access to PCa screening.

‘*This is the first of its kind for cancer doctors to come here. No one talks about cancer in prison! So, it is better to sensitize us, screen and treat us’*. (**FGD _Gulu Main Prison, above 54 years**)

‘*Medical workers need to come and attend to us so that we can know whether we have prostate cancer’* (**FGD Mbale Main Prison, above 54 years**)

‘*Bring the testing equipment and have collaboration with the cancer unit for services’* (**FGD_ Mbale Main Prison, 40-54 years**)

‘*Here thing works on order, so make use of the prison authorities to handle issues of prostate cancer screening’* **(FGD_ Luzira Prison, below 54 years)**

Furthermore, the availability of PCa screening equipment to the prison setting, coupled with training health providers to offer the screening services were looked at as ways of motivating men in Ugandan prisons to seek PCa screening. Similarly, having outreaches that target conducting PCa screening services by experts was another suggestion given in case the health units in prisons are not equipped yet.

‘*Train the staff and orientate the health workers’* (**KII**)

‘*If we get the knowledge about PCa, we can deliver the message unto them, whoever comes for medical attention we can screen them if we have the screening gadgets, we can do that. And whoever comes we sensitize, educate them about PCa, if they go back to the community, they can also pass the same information to their people to seek medical attention’* (**KII)**

‘Organize *the health workers of prisons to be able to handle the screening’* (**FGD_Luzira prison, above 54 years)**

Also, the inmates narrated that there is a need to improve the infrastructure in terms of space so that these services can be provided in an environment that can ensure privacy and confidentiality.

‘*We must be ready to have at least some areas which are free for screening’*. (**KII_**)

Introducing programme that target the population at risk of PCa for example creating awareness is another facilitator to PCa screening. Improving referral services for those that present with symptoms of PCa was also one way to motivate men in prison to take up the screening services

#### Intervention strategies for promoting PCa screening in prisons

Pertaining to the best way to educate or have information passed on to men in prison, a number of strategies were suggested, among which was to have PCa as one of the topics to be shared as part of the health education sessions in the prison. However, for this to be successful there is a need to orient the prison health workers so as to give a comprehensive package on PCa. More so, the use of visual IEC materials while conducting health education is required to give a clear understanding of the information that is being shared.

’*Here the education is a bit easier like when you use posters. You tell them where the problem is because not all of them understand English or native languages because we have people from different tribes’* (**KII)**

‘*Train the counselors to handle cancer related problems within the prisons systems’* (**FGD_Luzira Prison, above 54 years)**

‘*In Acholi (one of the tribes in northern Uganda), we say what you start, you have to finish it. This means for us we are looking at you doctors who have come here to start the process of helping us, sensitize us, screen and provide treatment for us to save us from cancer, including prostate cancer’* (**FGD _ Gulu Main Prison, 40–54 years**)

Moreover, the use of the existing structures in the prison setting particularly health providers within the prison setting was one strategy pointed out that would promote PCa screening. This requires the engagement of prison officers to spearhead the campaigns for screening services.

‘*Having this service spearheaded by the prison management to have the prisoners attend the screening services is very important. Any other? No, that is the best, actually I have pointed out the best way. Have the prison authorities, yes, and the prison health services to ensure prostate cancer screening is done in prison setting’*. (**KII**)

Similarly, there was a recommendation to have staff dedicated to conduct health education within the prison setting and one of their roles would be to talk about cancer, including PCa during parades because parades were believed to be one of the avenues that are used to pass information to the prisoners.

‘*Make use of our prison daily parades to inform the prisoners’.* (**KII**)

‘*We have health workers who do work but not on prostate cancer, so if you can introduce health workers amongst the schedule of duties, it’s the best because it is easier’*. (**KII)**

‘*The best way to come and help us is to come to the prison and sensitize the prisoners about the prostate cancer and check us’*. (**FGD_ Gulu Main Prison, above 54 year**)

‘*Assign medical staff to address the prisoners and the staff during the routine parade’* (**KII)**

Additionally, integrating PCa screening services in routine health services delivery like other services that have been introduced within the prison was perceived as one way of promoting PCa screening among men in prison as this would lead to easy accessibility. It was suggested that the integration should include comprehensive information about PCa to address the misconceptions surrounding PCa, including using the appropriate IEC materials.

‘*Other programs have been there like HIV testing, circumcision, and even testing of COVID 19, where the prisoners are allowed to go the prison health center. Now since this has come, it can be integrated alongside those which have been existing’*. (**KII**)

‘*Health education we normally embark on it in Outpatient department when they are coming for sickness, we also have peer groups in each ward, we have health monitors in each ward we have trained them, we have counselors we have trained them so those ones have that knowledge but lacking the knowledge of cancer we haven’t trained them about it so if we also equip these counselors about the knowledge of PC, I know we shall get them’* (**KII**)

‘*Giving them accurate information is crucial. Tell them how cancer is gotten, the possible causes, the consequences of getting cancer, and even tell them that there is hope even after being diagnosed with cancer. tell them services are available, in fact the thing that can entice them, make services free of charge and they will come very fast’*. (**KII**)

‘Need* sensitization of both prisoners and staff’* (**FGD _ Gulu Main Prison, 40–54 years**)

‘*Informing them the danger of PC is and how good it is to be screened. It is to know whether you have PC or not so that you can live a better life’.* (**KII**)

Still in line with using IEC materials, there was a call that the materials should be translated in the commonest local languages spoken in a given area, since prison setting have different ethnic groups and also for those that are not educated.

‘*We could get people we call counselors or people who come to teach prisoners. We should get books or handouts for those who know how to read English or Luganda and use the media’. (***FGD _Luzira prison, above 54 years)**

‘*If we have posters alerting them on the signs in places where they can easily excess, yeah, both in translated languages so that they can be able to know the message and then it can be made as part of the assemblies, they always talk to them, they converge them and always give them updates’* (**KII)**

In addition, there is a need to improve the referral process so that there is assurance to men in prison that in case one is diagnosed with PCa there is an opportunity to receive care.

‘*In case of anything found like somebody having an issue at least ease the referral to get treatment because just knowing and then you don’t have the capacity to seek medication is not helpful. It is as good as not doing it. Because here the rules remain rules in prison and they are like not easily shaken’* (**KII)**

**‘***The best way to make people check the people is if you find the disease, treat and cure it. This will make people to get motivated to screen while knowing that they can be cured’*. **(FGD_Gulu Main Prison, above 54 years)**

Nonetheless, the use of peer educators was believed to make the PCa information more acceptable to the inmates. In this case, peer educators would be men in prisons who are given a basic orientation to enable them to sensitise fellow men in prisons on PCa. This is believed to create a sense of trust since it is their fellow inmates as it has been used in other health programme and yielded good results, for example, in TB and HIV/AIDS programme.

‘*Prison services should accept Cancer Institute to come here to train trainees inside prisons because even for other diseases we have counselors who help us here like TB, HIV, and there are quick responses here. If you give them priority and chance then we shall be educated t and treated’*. (**FGD _Luzira prison Urban, above 54 years**)

‘*Have training of peer educators to help their colleagues can work. People believe in their friends especially the inmates they believe in their friends’* (**KII**)

‘*For this screening programme to work well, you need to work with peer educators like in the HIV/AIDS programme. This will increase access to prostate cancer awareness and screening in prison’*. (**FGD_Gulu Main Prison, 40–54 years)**

**‘**Use* peer educators by training them as we have already existing system within the prisons and use the prison authorities*’**(FGD_Luzira Prison, above 54 years)**

Similarly, some participants suggested that men in prison can be put into small discussion groups or one on one where information about PCa can be shared, this would also enhance the sense of confidentiality since PCa is considered a private issue.

*‘One on one information is also okay for us. You come sit with me and I talk to you, then I believe the information you are going to take won’t spread out’*. (**KII**)

Conducting outreaches in the prison setting was another strategy that was suggested to help men in prison to screen for PCa as this would make services easily accessed because the health units within the prisons do not have capacity to offer cancer-screening services.

‘*Me if at all the prison accepts competent doctors to come and check who has and who does not and it helps us to get early treatment or prevent it’*.** (FGD _Luzira prison, above 54 years)**

‘*We have no time to get out for cancer screening. Cancer doctors should come and screen us from here in prison’*. (**FGD_Gulu main, above 54 years**)

Similarly, one of the participants suggested that UCI and the prison management can have an arrangement to have men in prison have a programme where they are brought to the cancer institute or the regional cancer centre for checkups according to the schedule as required. This option was believed to be cost-effective compared to outreaches. More so, equipping the health facilities that are within or close to the prison setting would be another alternative where men in prison, especially those considered at risk can be supported to access screening.

‘*Screening services need to be brought near to allow access to screening at least once a year*’ (**KII**)

‘*It is better when you make programme for routine checkup in prison just like the HIV testing programme’*. (**FGD_Gulu main prison, 40–54 years**)

‘*Still of course it involves both parties, prison management and the Uganda Cancer Institute management………. because it involves more resources as compared to when prisoners come to the cancer centre’*. (K**II**)

The use of mass media was another suggestion, it was found that men in the urban prisons were reported to have access to TVs and radios in common places, therefore, this can be one strategy through which health information pertaining to PCa can be passed.

‘*We have TV screens, if we are given electronic information, we can watch them. In fact, these wards have TVS. Almost everybody has got a screen in their wards. Through that we can plan so that each ward gets a chance of being educated’*. (**KII**)

‘To address lack of *awareness, TV, posters and brochures need to be used to create awareness’* (**FGD_ Luzira Prison, 40–54 years)**

### Quantitative results

#### Social demographic characteristics of the participants

Out of the planned the sample size of 2,863, this study enrolled a total of 2,565 participants for the survey (Table 1). The mean age was 50.2 (9.8), the majority were aged 40 to 54 (74.4%) compared to the participants who were aged 55 years and above, most were married (74.4%) and least educated (none and primary level education (72.1%), and the majority were peasant farmers before becoming prisoners (53.7%), and only 1.2% screened when in prison (Table 1).

#### Men’s perceptions regarding PCa screening

**Figure 1.**
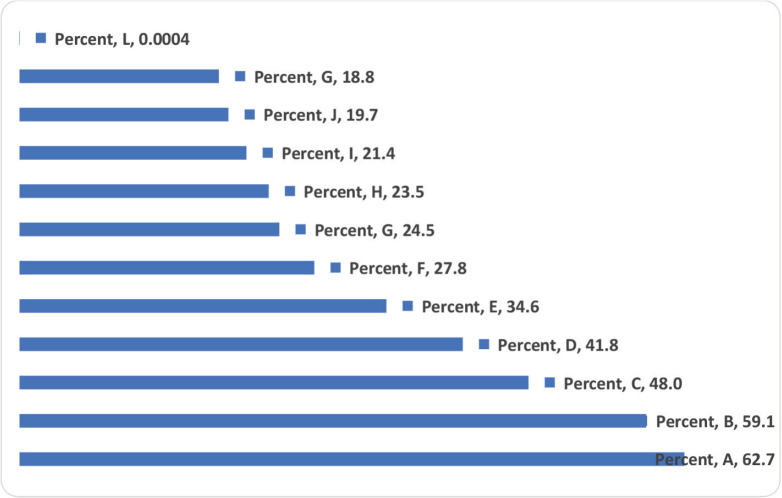
What men perceive of PCa screening (Multiple responses). A: Better make it mandatory for all men of the eligible age group to check for PCa. B: PCa is like any other diseases that require the patient to contact a doctor immediately. C: It requires regular check-up/testing, and providing treatment for those found with PCa. D: Prisoners do not know the actual cause of PCa. E: A common type of cancer in males, that begins in the prostate gland and can be caused by genetic or lifestyle risk factors. F: It is what makes men in prisons urinate frequently. G: It is caused by infections acquired from the unhygienic prison bathrooms and toilets. H: Prisoners think it is a sexually transmitted disease. I: It is caused by eating bad and monotonous food. J: It affects more men who are not sexually active, like men in prisons. K: It is caused by homosexuality. L: Other.

**Figure 2.**
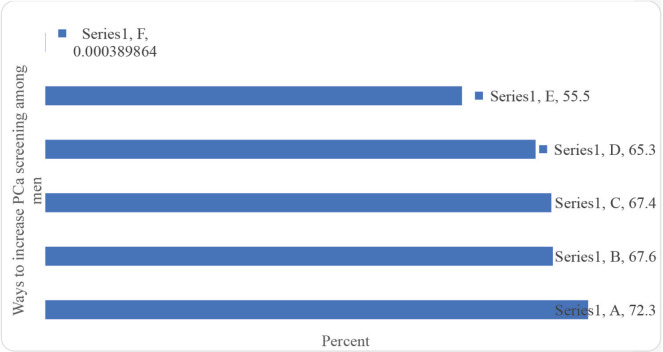
Ways to increase PCa screening (Multiple responses). A: Create awareness. B: Cancer Centres/hospital should conduct screening outreach in prisons. C: Government should provide equipment for PCa screening in prisons' health facilities. D: Train medical staff in prison health centres on PCa screening. E: Screen men in prisons for PCa regularly. F: Other.

Regarding perception about PCa screening, most felt that it would be better to make it mandatory for all men of the eligible age group to check for PCa. Also, most indicated that PCa should be viewed like any other diseases that require the patient to contact a doctor immediately and it requires regular check-ups, and provision of treatment for those found with PCa (Figure 1). Creating PCa awareness, conducting screening outreach in prisons and providing equipment for PCa screening in prisons health facilities were viewed by the majority as ways to increase PCa screening (Figure 2).

#### Motivators for PCa screening

**Figure 3.**
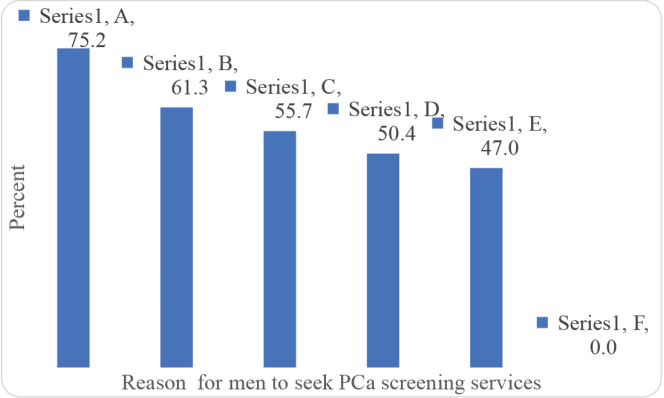
Reasons for men to seek cancer screening (Multiple responses). A: When I feel pain while urinating. B: If treatment is free for the prisoners diagnosed with PCa. C: If PCa screening services are available in prisons. D: When experiencing frequent urination. E: When not in control of my urination. F: Other.

**Figure 4.**
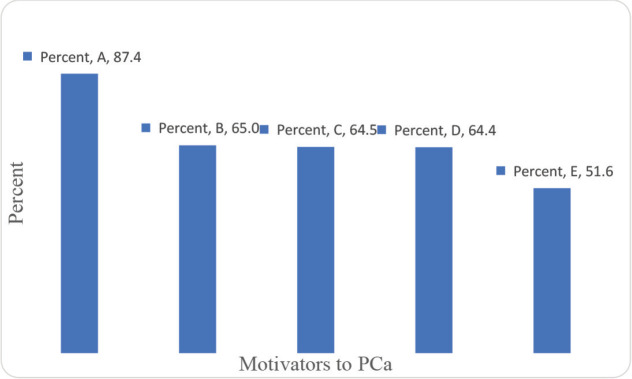
Motivators to PCa screening. A: Make services for PCa screening free of charge. B: Provide counselling services to the prisoners. C: Train the prison health workers on PCa screening. D: Create awareness on PCa. E: Use peer educators.

The majority of the participants indicated that what might drive them to seek PCa screening are; when they feel pain while urinating, if treatment is free for the prisoners diagnosed with PCa, and if PCa screening services are available in prisons (Figure 3). In addition, making services for PCa screening free of charge and provision of counselling services to the prisoners were viewed by the majority as the main motivators for PCa screening (Figure 4).

#### Barriers towards PCa screening

**Figure 5.**
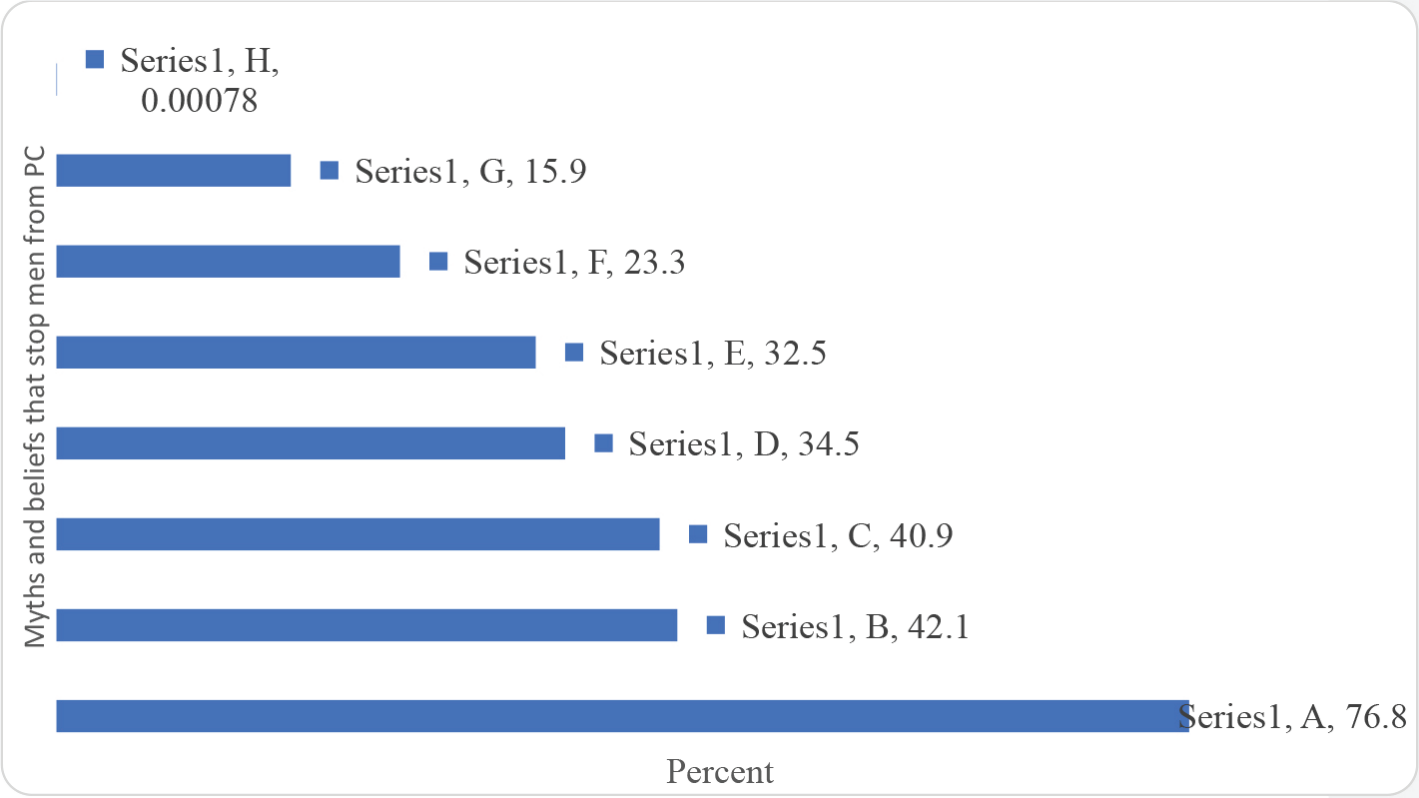
Barriers to PCa: myths and beliefs that influence screening practices (Multiple Responses). A: People fear medical check-up around their private parts. B: If men are checked and found with no PCa, they think it is a waste of money and time. C: Men do not have time for their health check-ups. D: PCa biopsy (sample removal to confirm the cancer) spread the cancer and makes it fatal. E: There is no cure for PCa. F: PCa diagnosis means deaths. G: PCa is caused by witchcraft. H: Other.

The barriers to PCa screening were mainly myths and beliefs that may influence PCa screening practices. The fear for medical check-ups around the men’s private parts, if checked and found with no PCa, they think it is a waste of money and time, men do not have time for their health check-ups, and fear that PCa biopsy (sample removal to confirm the cancer) spread the cancer and makes it fatal were the major barriers reported by the participants (Figure 5).

#### Intervention strategies for promoting PCa screening

**Figure 6.**
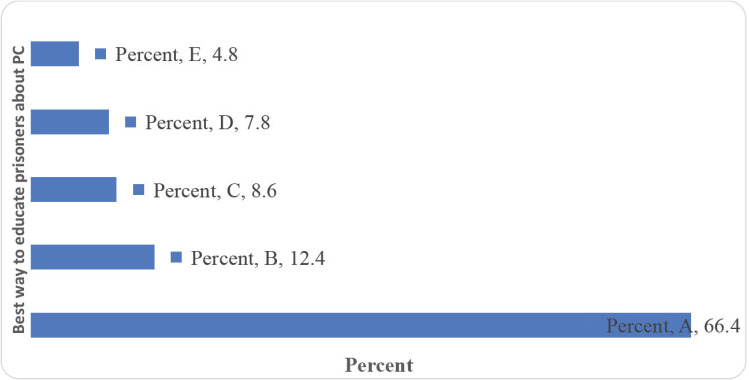
What is the best way to educate men in Uganda prisons about PCa? A: Give prisons some PCa information booklets/posters. B: Conduct group health education sessions. C: Conduct PCa awareness on radios and/or TVs. D: Use peer educators. E: Use daily prison parades.

**Figure 7.**
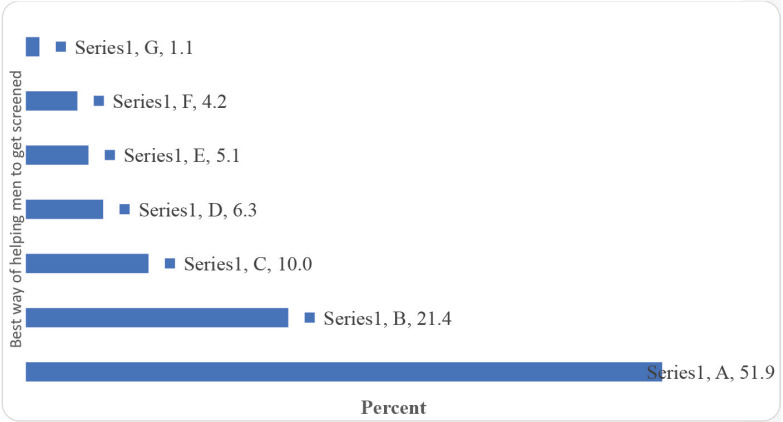
What is the best way of helping men in Uganda prisons to get screened for PCa? A: Train the prison medical staff on how to screen for PCa. B: Equip prison health facilities with PCa screening supplies. C: Work with the UPS to train and equip Prison Health Centres to screen for PCa. D: Provide continuous and targeted counselling and guidance. E: Include all men, women and all ages screening PCa activities. F: Screen eligible men for PCa at admission. G:Conduct outreach from the cancer hospital.

Giving the inmates PCa information booklets/posters and conducting group health education sessions were viewed as some of the best ways to educate men in Uganda prisons about PCa (Figure 6). Besides, by majority, the best way of helping men in Uganda prisons to get screened for PCa was found to be training the prison medical staff on how to screen for PCa, equipping the prison health facilities with PCa screening supplies, and working with the UPS to train and equip Prison Health Centres to screen for PCa (Figure 7)

## Discussion

Several key items emerged as important considerations for planning potential interventions to improve access and adherence to PCa screening among the male inmates in Ugandan prisons. From the FGDs and KIIs, PCa was perceived as a disease that makes men urinate frequently, caused by bad monotonous prison food, caused by infections got from the unhygienic prison bathrooms and toilets and affect more men who are not sexually active, like men in prisons. Although some participants had a positive perception that early detection for PCa is very important, majority did not have any value attached to early detection since they had a perception that PCa is an incurable disease which would lead to death irrespective of whether it is detected early. Therefore, factors that should be taken into consideration in developing and planning interventions to promote PCa screening among men in Ugandan prisons include perceptions about PCa, in particular, the fatalistic beliefs held by most of the inmates.

Creating awareness of PCa was more stressed to be an important aspect towards PCa early detection as most of the men were not knowledgeable about the disease. The general myths/belief that all cancers have no cure was one barrier that inhibited most participants not to consider screening as of any value, coupled with fear of being told that one is positive and the stress that comes with it. Participants revealed that they would rather live without getting to know the unknown. In addition, lack of knowledge of PCa and lack of Pca screening services in prisons were perceived as barriers to PCa screening in prison settings**.**

A review of both published and unpublished data shows that there is no information about barriers to and facilitators of PCa screening in prison settings. However, a few studies on cancer in prison settings have been conducted, only in Australia, Canada, and the United States of America, the most recent being a review study by Olds *et al* [12]. This scoping review reported limited research on barriers to cancer services access in prison settings and further recommended the need to carry out more studies to effectively elucidate how barriers to good quality cancer care for prisoners may be identified and mitigated. Nevertheless, similar to some of our findings, studies among the African Americans [21, 22] also found that lack of awareness and knowledge, negative beliefs and fears, and seeking healthcare only when symptoms appear, were barriers to PCa screening.

In our study, suggestions by the study participants on how awareness would be created included the use of peer educators among inmates who should give basic information on the screening and PCa, use of IEC materials. On what could be the facilitators to increase PCa screening, most of the participants said that they would have themselves screened if the government comes up with the programme for PCa screening for the prisoners, provide cancer specialists and the testing facilities within the prisons or nearby, and backed by awareness about the PCa. Availability of equipment to the prison setting coupled with training healthcare providers to offer the services was looked at as one way of motivating cancer screening among men in prison. Similarly, having outreaches that target conducting PCa screening services by cancer experts was another suggestion given in case the health units in prisons are not equipped yet.

Overall, qualitatively, the interventional strategies that men in Uganda prisons believed could help health care providers to promote and increase PCa screening were; using the prison peer educators, working with the UPS to train and equip prison Health Centres to screen for PCa, conducting outreach from the cancer hospital, MoH to work with the UPS authority to establish a system for PCa screening in prisons, using health workers within prisons, using the regional prisons health centres to establish PCa screening in prisons, using IEC materials such as posters, and conducting group health education sessions.

Similarly, the survey that enrolled a total of 2,565 participants with a mean age of 50.2 (9.8), indicated that most male inmates felt that it would be better to make PCa screening mandatory for all eligible men in prison. Also, most indicated that PCa should be viewed like any other disease that requires the patient to contact a doctor immediately and it requires regular check-u, and provision of treatment for those found with PCa. Creating PCa awareness, conducting screening outreach in prisons, and providing equipment for PCa screening in prison health facilities were viewed by the majority as ways to increase PCa screening.

Majority of the participants indicated that what might drive them to seek PCa screening are; when one feel pain while urinating, if treatment is free for the prisoners diagnosed with PCa, and if PCa screening services are available in prisons. In addition, making services for PCa screening free of charge and provision of counselling services to the prisoners were viewed by the majority as the main motivators for PCa screening.

The barriers to PCa screening were mainly myths and beliefs that may influence PCa screening practices. The fear of medical check-ups around the men’s private parts, if checked and found with no PCa, they think it is a waste of money and time, men do not have time for their health check-ups, and fear that PCa biopsy (sample removal to confirm the cancer) spread the cancer and makes it fatal, besides limited access to health workers recommendation, were the major barriers reported by the participants. Therefore, consideration should be given to developing interventions to increase awareness among the inmates and the healthcare professional regarding the importance of their role both as a barrier and facilitator to PCa screening among men in prison settings. Meanwhile, though not in a prison setting, in another survey in the USA, greater worry about PCa and screening fear were the significant barriers among the African American and African-Caribbean men. On the other hand, access to health workers and annual visits to health facilities were the main facilitators of PCa screening using DRE among the African American and African-Caribbean men [23].

Moreover, our findings corroborate with another non-prison setting study in the USA, which examined the perceptions, attitudes, and beliefs regarding barriers and facilitators to PCa screening among Filipino men in Hawaii, using exploratory, qualitative design [24]. From the USA qualitative study, the barriers to PCa screening included a lack of awareness of the need for screening, reticence to seek healthcare when feeling well, fear of cancer diagnosis, financial issues, time constraints, and embarrassment. The same study found that the presence of urinary symptoms, personal experience with family or friend who had cancer, and receiving recommendations from a healthcare provider regarding screening were facilitators for screening the same study found that the potential culturally relevant interventional strategies to promote PCa screening included screening recommendations from health professionals and cancer survivors; radio and television messages, and newspaper articles targeted to the Filipino community; informational brochures; and interactive, educational forums facilitated by Filipino multilingual, male healthcare professionals [24].

Also, besides the influence of prison setting, lower socioeconomic status, less PCa knowledge, and lack of health workers' recommendation for screening [25]. The fear and anxiety related to PCa and the screening process can also represent key barriers that are potentially modifiable. Although men might undergo screening because of their decreased PCa fear, inversely, they could also avoid screening to avoid an unfavourable diagnosis or embarrassment regarding the test itself, especially with DREs [26]. For instance, in another study by Consedine *et al* [27], the fear of screening, especially the embarrassment and discomfort regarding DREs, was a significant barrier to PCa screening, but the PCa fear was a facilitator to screening. Therefore, concern and fear might follow a bimodal distribution, with moderate levels promoting health behaviours and high levels promoting denial and avoidance [27–29].

Therefore, there is a need to be aware of the increased health needs, including the unmet needs for cancer control especially cancer screening among the older prisoners during incarceration. An understanding of the possible barriers and the facilitators is paramount in guiding health programme implementation. As such, prevention of health deteriorations, including cancer prevention and early detection, advocacy for more seamless health care in prison settings and when the incarcerated offenders transition to the community are crucial [30].

Overall, giving the inmates PCa information booklets/posters and conducting group health education sessions were viewed as some of the best ways to educate men in Uganda prisons about PCa. Besides, by majority, the best way of helping men in Uganda prisons to get screened for PCa was found to be training the prison medical staff on how to screen for PCa, equipping the prison health facilities with PCa screening supplies, and working with the UPS to train and equip Prison Health Centres to screen for PCa. In terms of the implications for practice, policy and future research, consideration should be given to developing interventions to increase awareness among both the inmates and the healthcare professional regarding the importance of their role in facilitating PCa screening among men in prison while ensuring that the prison health facilities are equipped with the required screening logistics.

### Study strengths and limitations

The explanatory sequential mixed methods design approach employed in this study gives it more strength compared to studies that solely used either qualitative or quantitative approaches. Also, participants from both urban and rural prisons were included in this study, thus, increasing the degree of generalizability to men in prison population. The limitation of this study lies in the general weakness associated with cross-sectional studies such as susceptibility to misclassification due to recall bias and depicting the health-related state or event and other variables of interest as they exist in a defined population at a single point in time.

## Conclusion

Culturally relevant interventions are needed to address the barriers to PCa screening among men in Ugandan prisons. Therefore, consideration should be given to developing interventions to increase awareness among both the inmates, the prison health system, including the healthcare professional regarding the importance of their role in facilitating PCa screening among while ensuring that the prison health facilities are equipped with the required screening logistics.

## Authors’ contributions

Dr Jackson Orem (UCI), the principal investigator of this study, contributed to the overall project design, management, and manuscript writing. Prof. Lynn Atuyambe (Makerere University, School of Public Health) supervised the study and gave insight on the qualitative data analysis, and manuscript writing. Jatho Alfred (UCI and King Caesar University) developed the data collection tools, sought for the study approval, supervised data collection and analysis, and drafted the manuscript. Prof. Annabella HabinkaBasaza-Ejiri (King Ceasar University) supervised the study and gave insight into the quantitative data analysis, and manuscript writing. Innocent Atuhe (King Caesar University and Uganda Cancer Fund) conceptualised the study, contributed to the project management, and manuscript writing. Babra Nalwadda (Uganda Cancer Fund) contributed to data collection and manuscript writing.

## Funding

This study was funded by Prostate Cancer Foundation, through Pfizer, grant number 67651829. No financial relationships to disclose.

## Conflicts of interest

The authors have declared no conflict of interest.

## Data availability statement

All relevant data are within this paper.

## Figures and Tables

**Table 1.** Social demographic characteristics of the participants.

Variables	Number (Percent)
Sample size	2,565 (100.0)
Study site (Region)	
Central	890 (34.7)
East	629 (24.5)
West	526 (20.5)
North	520 (20.3)
Site district	
Kampala, Central, Central/Buganda	691 (26.9)
Mbarara City, Western, South Western	526 (20.5)
Gulu City, Northern, Acholi	394 (15.4)
Soroti City, Eastern, Teso	341 (13.3)
Mbale City, Eastern, Elgon	240 (9.4)
Masaka City, Central, Central/Buganda	199 (7.8)
Oyam, Northern, Lango	126 (4.9)
Budaka, Eastern, Teso	48 (1.9)
Study site (Name of prison)	
Luzira MB	691 (26.9)
Gulu Main	394 (15.4)
Mbarara Main	373 (14.5)
Soroti Main	341 (13.3)
Mbale Main	240 (9.4)
Masaka Main	199 (7.8)
Kakiika	153 (6.0)
Loro	126 (4.9)
Budaka	48 (1.9)
Study site setting	
Urban	2,391 (93.2)
Rural	174 (6.8)
Age in complete years	
Mean (SD)	50.2 (9.8)
Median (IQR)	47.0 (12.0)
Median (IQI)	47.0 (43.0; 55.0)
Age group in complete years	
40 to 54	1,909 (74.4)
55 and above	656 (25.6)
Highest level of education?	
a) None	552 (21.5)
b) Primary	1,366 (53.3)
c) Secondary	485 (18.9)
d) Tertiary	162 (6.3)
Religion	
f) Orthodox	9 (0.4)
e) Seventh day adventist others	67 (2.6)
c) Muslim	362 (14.1)
d) Pentecostal/born-again faith	401 (15.6)
b) Anglican (Protestant)	569 (22.2)
a) Catholic	1,156 (45.1)
g) Others (Specify)	1 (0.0)
Marital status	
b) Married	1,909 (74.4)
a) Single	357 (13.9)
c) Divorced or separated	197 (7.7)
e) Widowed	102 (4.0)
Highest level of education obtained by your partner *n*	
a) None	408 (18.2)
b) Primary	1,205 (53.9)
c) Secondary	515 (23.0)
d) Tertiary	109 (4.9)
Occupation before becoming a prisoner	
c) Peasant farmer	1,377 (53.7)
b) Business owner	713 (27.8)
d) Employee (organizational/company staff, teacher, nurse, doctor, engineer, etc.)?	346 (13.5)
a) Student	94 (3.7)
e) Other, specify	35 (1.4)
Engaged in a certain occupation in this prison	
b) No	1,659 (64.7)
a) Yes	906 (35.3)
If engaged in a certain occupation in the prison, current occupation	
Handcrafts	311 (34.6)
Farming	267 (29.7)
Teaching/lecturing	103 (11.4)
Carpentry	97 (10.8)
Tailoring	81 (9.0)
Other, specify	41 (4.6)
Region of origin?	
c) Eastern	778 (30.3)
d) Western	675 (26.3)
b) Northern	578 (22.5)
a) Central	533 (20.8)
e) Non-Ugandan, specify	1 (0.0)
Residence area before becoming a prisoner?	
a) Rural	1,697 (66.2)
b) Urban	688 (26.8)
c) Urban slum	114 (4.4)
d) Peri-urban	66 (2.6)
Ever screened for PCa before becoming a prisoner?	
Yes	59 (2.3)
No	2,506 (97.7)
Ever screened for PCa when in a prison?	
Yes	32 (1.2)
No	2,533 (98.8)
